# Society Builds Sustainability in Africa

**DOI:** 10.1289/ehp.115-a246

**Published:** 2007-05

**Authors:** Tanya Tillett

With the speed of industrialization in today’s global community, the costs of disparities in environmental health and risk assessment can be dangerously high in developing countries without broad, stable regulatory and protective measures in place. Addressing capacity-building problems will depend largely upon the implementation of proactive measures within the borders of these developing nations—measures that participants sought to create at the Risk Assessment and Quality Assurance Training Workshop of the African Society for Toxicological Sciences (ASTS), held 21–28 October 2006 in Limbe, Cameroon.

The workshop was cosponsored by the NIEHS as part of its efforts to expand global environmental health initiatives, as outlined in the institute’s Strategic Plan. Forty-seven experts in toxicology research, environmental policy, and government from Cameroon, Nigeria, Sudan, South Africa, the United States, and Europe convened for the purpose of generating ideas for new sustainable development initiatives. Attendees also took part in training modules and a site visit to a local oil refinery.

Sanmi Areola, a toxicologist with the Metro Nashville/Davidson County (Tennessee) Public Health Department and incoming ASTS president, says that organizations such as the ASTS serve as necessary bridges of communication between developed and developing nations. “Continuing and emerging environmental [and] public health issues present differently in Africa compared to the developed countries of the world primarily because of the lack of enforceable policies and regulations and the nonexistence of infrastructures, [which are] poor where and when they exist,” says Areola. He explains that the negative impacts of environmental stressors on public health in Africa are exacerbated by poverty, political instability, urbanization, and overpopulation, among other factors. “These issues must be addressed through a multifaceted, multidisciplinary, region-specific approach where the identification of hazards and characterization of the risks take into consideration the uniqueness of the African geopolitical and ecological divides,” he says.

According to Areola, the ASTS is uniquely positioned to provide a platform and serve as the facilitator for a collaborative partnership with agencies from developed nations to build approaches for managing and alleviating these risks. In the past 10 years, the ASTS has built solid networking structures, working with policy makers, scientists, and agencies within and outside Africa.

Outgoing ASTS president Hoffman Moka Lantum, director of practice variance with Excellus BlueCross BlueShield, agrees, saying that a multidisciplinary, international exchange of ideas has been and will continue to be integral to the success of the ASTS’s efforts. He points out that very few data are based on studies done in Africa despite exponential growth in the use of large-volume chemicals in the petrochemical, mining, agrochemical, textile, and food industries, plus disproportionate underlying disease and nutritional disorders from food deficiency and toxicity. “The effects of the chemical burden from imported new classes of drugs, detergents, and industrial hydrocarbons on the biology and ecology of Africa are largely unknown and unappreciated, and may never be talked about if our colleagues in developed countries do not participate in this [ongoing] discussion,” he says.

Kenneth Olden, a founding member of the ASTS and past director of the NIEHS and the National Toxicology Program, says all parties can benefit from such collaborative efforts. “Environmental health issues are national in scope, so it is important that nations, including the United States, cooperate in research, training, and exchange of prevention and remediation technology. All nations, including the African nations, have much to contribute to environmental protection,” he says.

## Identifying Areas of Need

This emphasis on the sharing of information across disciplines and cultures led to a highly interactive dialogue between the workshop participants concerning how best to address the vulnerability of Africans to increasingly complex scenarios involving potentially harmful chemical exposures. One key component of these scenarios is gene–environment interaction.

Olden, who served as honorary cochair of the workshop, says the gene–environment connection plays a fundamental role in human health outcomes, and it is therefore important to implement large-scale, regulated environmental health safeguards in African countries to address the public health disparities between those nations and more affluent countries. “Since all humans are virtually identical with respect to genetics, the environment—which includes diet, lifestyle, poverty, and its consequences—is the major contributor to differences in health and disease between the U.S. and African populations,” Olden explains. “So the biggest pay-off in terms of prevention and intervention would be in the area of environmental protection and remediation.”

But how can regulation and remediation stay abreast of the accelerated environmental and industrial changes occurring in Africa? In many African countries, urbanization is rapidly outpacing capacity. Cities are becoming increasingly overcrowded, and in recent years, there has been an influx of imported electronics such as computers and televisions. But with scarce means to safely dispose of these items once they are discarded, the people of these countries are being exposed to potentially dangerous chemicals when electronics are simply dumped or burned (for more information, see “Unfair Trade: e-Waste in Africa,” *EHP* 114:A232–A235 [2006]).

Exposures to chemicals in common household products, petroleum product spillage, pesticides, and easily accessible counterfeit pharmaceuticals also pose health threats. According to the preliminary proceedings of the workshop, one of the three main objectives was to address these hazards by determining key elements of sustainable policies and practices in Africa regarding the use and marketing of industrial raw material, chemical products, finished goods, and waste.

Lantum says it is critical to promote awareness of local, regional, and worldwide issues pertaining to household and industrial use of chemicals in Africa. These exposures could grow into a much larger problem if more attention is not paid to heading them off, he warns. “This is a risk that far outweighs the effects of HIV/AIDS, malaria, and malnutrition,” he says. “Chemicals in our environment are crucial to how the body will react to infections and other diseases. Lack of clean water, soil and groundwater pollution, and poor indoor and outdoor air are existing problems that can easily develop into epidemics of chemical exposure if proper use and disposal of chemicals is not made a priority. We need a thorough understanding of the true potential of household and industrial chemicals to enhance the disease burden in Africa.”

Areola says that chemical threats to environmental health in Africa escalate when combined with existing problems. “We must consider overlapping issues of environmental risk, demographic, and epidemiological transitions; unlike many developed countries, African nations are still at the basic stages of these transitions,” he explains. “Consequently, emerging risks from industrial pollution, chemical wastes, pesticide usage, and illegal dumping of industrial wastes overlap existing environmental issues such as inadequate solid waste management, environmental sanitation, and wastewater management.”

Workshop attendees agreed that more internal and international resources should be identified so that adequate reproducible quality assurance measures could be implemented. The conference training modules stressed the importance of creating a solid foundation for data collection and distribution. One, for example, focused on proper classification and labeling of chemicals for transport or supply based on international regulations such as the UN recommendations for its Globally Harmonized System of Classification and Labelling of Chemicals.

The participants also received training in standard testing protocols and new molecular methodologies for hazard characterization of chemicals. Module presentations by African-based experts featured information on toxic metals such as lead and mercury, and also described the need for more information on the chemical composition of traditional medicines as well as possible toxicants that might contaminate them. This was seen as an especially pertinent issue given that more than 75% of inhabitants in African countries are believed to use traditional medicines.

The attendees also exchanged ideas on creating opportunities for human capacity building, particularly for researchers, industry experts, and government officers involved in chemical risk management. Course content at the workshop included training on the basics of human and environmental risk assessment, basic training in quality assurance, and case studies on health, safety, and environmental projects conducted in Africa. All African-based scientists who attended and completed quality assurance training at the workshop received a one-year membership to the Society of Quality Assurance, a Texas-based professional organization. “These participants are now empowered and engaged advocates for quality data collection and reporting. These are the kinds of partnerships that the ASTS seeks to facilitate,” says Lantum.

## Formulating a Plan of Action

Participants said African leaders should secure three key resources in order to establish robust, reliable, self-sustaining risk assessment and quality assurance programs. First, access to chemical information databases would make computational risk assessment and chemical analysis more affordable. Second, stable national, regional, and international collaborative risk assessment and quality assurance networks to help govern and control the process by which chemicals are imported into and exported out of African countries would help serve as the first line of defense against potentially harmful chemical exposures. Third, an educational center of excellence would provide practical, hands-on training in applied toxicological testing and experimentation for African-based experts.

In addition, grassroots activities and local mobilization are seen as especially important for maintaining momentum for the proposed initiatives on environmental health policy. During the meeting workshop participants formed the Cameroon Society for Toxicological Sciences (CSTS), with the ultimate goal of bringing industry, government, and researchers together with citizens to create sustainable social action programs on a local level.

Four immediate objectives for the CSTS were described to advance a public agenda for environmental health issues in African countries. The group pledged to establish a knowledge base of chemical sources that are prime suspects in environmental and public health effects in Cameroon and surrounding countries; create a computational health and environmental risk assessment network of experts to provide advice on the hazards of prime suspect chemicals to researchers, government regulators, and environmental specialists; publish papers from collaborative scientific research initiatives throughout the region in peer-reviewed journals; and develop and launch school-based social action programs to disseminate basic information on the potential environmental hazards related to household chemicals. “The CSTS allows for bidirectional exchange of information so that our goals and priorities are vetted by our colleagues based in Africa,” notes Lantum. “It will also be an appropriate body to translate global knowledge into local policy.”

## Moving Forward

Lantum says that more lobbying should be done for the establishment of robust and reliable networks of local or regional training, testing, and research infrastructure. “Risk assessment training using classical and modern computational methodologies must occur in Africa,” he stresses. If African scientists are able to develop their own policies and solutions, they will be able to implement them more successfully because they will have an inherent understanding of the problems unique to or more common in African countries. At the same time, participants agreed that development of strategies to detect and protect against potentially hazardous chemicals in the environment will benefit from information sharing with international colleagues who possess extensive toxicological experience.

Many African countries have adopted regulatory guidelines for the transportation, use and disposal of chemicals—guidelines that were developed in industrialized countries. Yet high costs and expensive technologies needed to enforce international protocols render most guidelines unenforceable for most African countries.

Lantum says, “Governments in Africa can learn from industrialized countries but are ultimately responsible for promoting sustainable development through the safe use of industrial and household chemicals. Adequate and enforceable regulations for the safe use of chemicals in African countries must be developed by local governments based on available resources. Partnerships with international research and policy institutions such as the NIEHS can be leveraged to complement local efforts and supplement the resources needed for robust chemical safety programs developed in less industrialized countries.”

With the continued fostering and development of international partnerships with the NIEHS and other like-minded organizations in developed nations, Areola sees a productive future for the ASTS as it seeks to address environmental health issues in African countries. He says, “Agencies such as NIEHS, with a publicized commitment to global health, are invited to work with ASTS to develop a roadmap for shaping policies and regulations for environmental risk management and to establish centers of excellence in Africa to provide continuing education to scientists in Africa.”

## Figures and Tables

**Figure:**
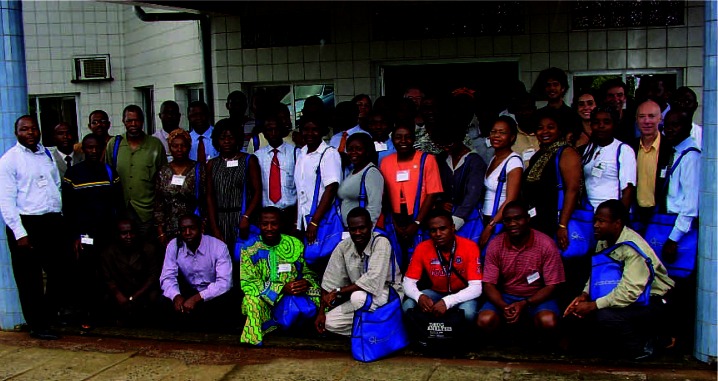
Take-home message Training in risk assessment and data quality assurance gives African professionals like these participants at an ASTS workshop in Limbe, Cameroon, the tools to build scientific capacity in their home nations.

